# Isolation of *Mycobacterium talmoniae* from a patient with diffuse panbronchiolitis: a case report

**DOI:** 10.1186/s12879-021-05944-9

**Published:** 2021-03-10

**Authors:** Tomoko Suzuki, Miwako Saitou, Yuriko Igarashi, Satoshi Mitarai, Katsunao Niitsuma

**Affiliations:** 1 Department of Infectious Disease and Pulmonary Medicine, Aizu Medical Center, Fukushima Medical University, 21-2 Maeda, Tanisawa, Kawahigashimachi, Aizuwakamatsu, Fukushima, 969-3492 Japan; 2 Department of Mycobacterium Reference and Research, The Research Institute of Tuberculosis, Japan Anti-tuberculosis Association, Tokyo, Japan

**Keywords:** Non-tuberculous mycobacteria, *Mycobacterium talmoniae*, Standard combination therapy, Drug-susceptibility, Diffuse panbronchiolitis

## Abstract

**Background:**

*Mycobacterium (M) talmoniae* isolated from a patient with cystic fibrosis was first described in 2017, and cases of *M. talmoniae* remain exceedingly rare.

**Case presentation:**

A 51-year-old woman had respiratory symptoms for 10 years. Diffuse panbronchiolitis (DPB) was detected at the first visit at our hospital. A cavity lesion in the apex of the left lung was found, and sputum and bronchoalveolar lavage fluid were acid-fast bacillus (AFB) smear- and culture-positive besides *Pseudomonas aeruginosa*. *M. talmoniae* was finally identified, and the standard combination therapy for non-tuberculous mycobacteria (NTM) was administered for 2 y referring to the drug-susceptibility test. Thereafter, the AFB culture was negative, the wall thickness of the lung cavity was ameliorated, and oxygen saturation improved.

**Conclusions:**

We encountered a rare case of *M. talmoniae* with DPB, for which standard combination therapy was effective. *M. talmoniae* may be considered a potential pathogen of lung disease, especially in patients with bronchiectatic lesions.

## Background

While the number of tuberculosis cases are decreasing, the increase in non-tuberculous mycobacteria (NTM) cases has become a global problem. NTM are environmental organisms that are frequently isolated from soil and water [1]. Although over 180 species have been discovered to date, only several species are reported to cause pulmonary disease [1]. The most commonly isolated species are the *Mycobacterium avium* complex (MAC), the *M. abscessus* complex (*M. abscessus* subsp. *abscessus*, *M. abscessus* subsp. *massiliense*, *M. abscessus* subsp. *bolletii*) [1]. The *M. fortuitum* group is a rare cause of pulmonary disease except in cases of achalasia and lipoid pneumonia [2, 3]. *M. gordonae* most often represents tap water contamination [4]. In Japan, a recent study showed that MAC accounts for nearly 90% of all NTM cases [5].

*M. talmoniae* was first described in 2017 and was isolated from human respiratory samples in the United States (US) [6]. It was classified as the most basal species of the slow-growing *Mycobacterium* clade [6]. In 2019, three cases of *M. talmoniae* isolation were reported in the US, and all patients had bronchiectatic lesions as underlying disease including cystic fibrosis (CF) [7].

Diffuse panbronchiolitis (DPB), which was first reported in Japan in 1969 [8], is mainly found in East Asia [9]. Although small numbers of case reports have recently been described, DPB remains a rare disease in Western countries excluding cases among Asian immigrants [10, 11]. The cause of DPB is unclear, but the disease is characterized by chronic inflammation in respiratory bronchioles and sinobronchial infection [12]. Immunogenetic studies revealed an association with human leukocyte antigen (HLA)-B54 in Japanese and HLA-A11 in Koreans [13]. Although low-dose, long-term erythromycin therapy for DPB, the effect of which is mainly attributed to an anti-inflammatory action, has drastically improved the survival rate [14, 15], DPB patients generally suffer from causative pathogens including *Pseudomonas aeruginosa* [13] and NTM, particularly MAC, *M. kansasii*, and *M. chelonae* [16]. Infections by these pathogens are related to reduced mucociliary clearance with bronchiectasis, which are characteristic findings in DPB and pathologically similar to CF.

This current report showed the rare case of *M. talmoniae* combined with DPB including the course of treatment for NTM.

## Case presentation

**Fig. 1:**
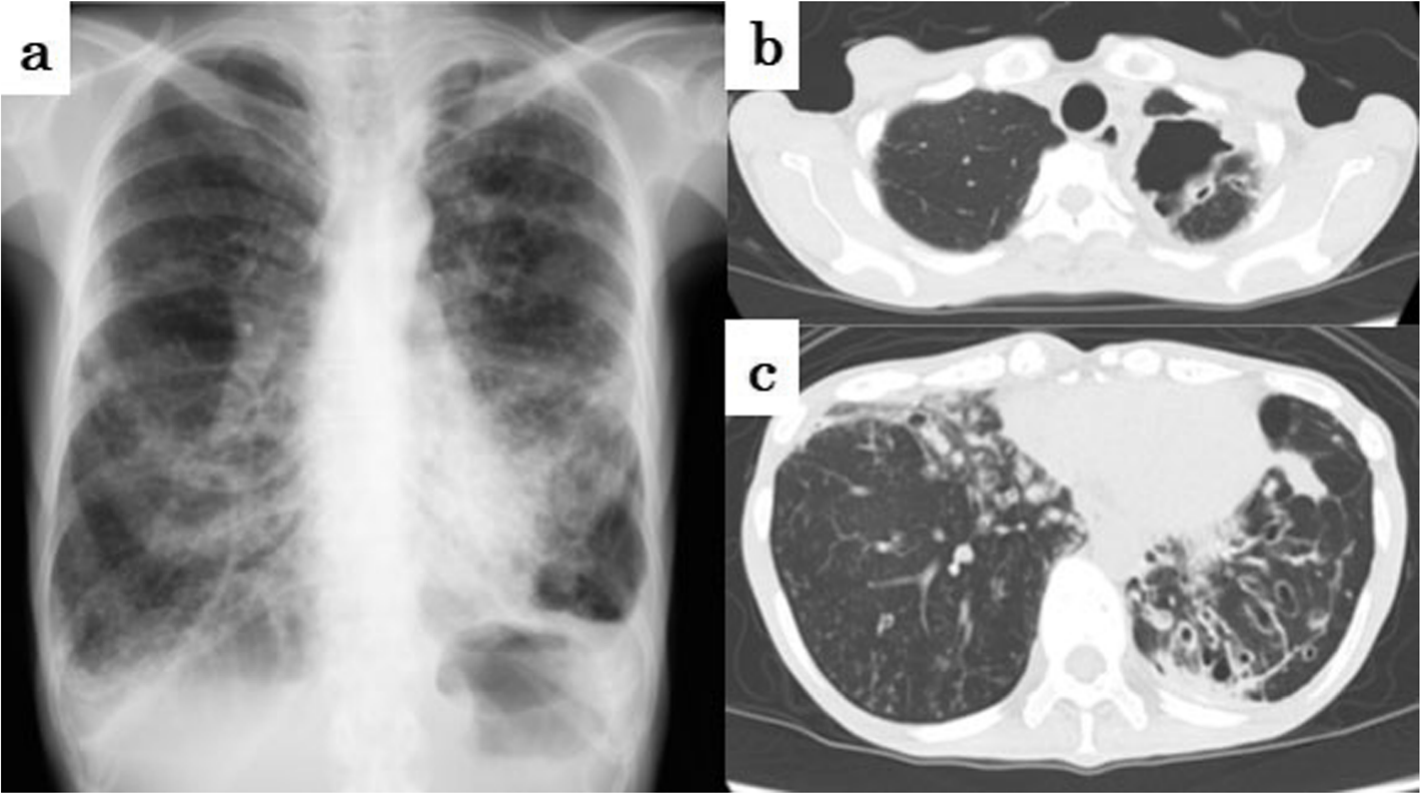
Chest images taken at the first visit at our institution in 2014. **a** Chest radiograph, **b** cavity lesion of the left lung apex on high-resolution computed tomography, and **c** left dominant bronchiectatic lesions on high-resolution computed tomography are shown

**Fig. 2:**
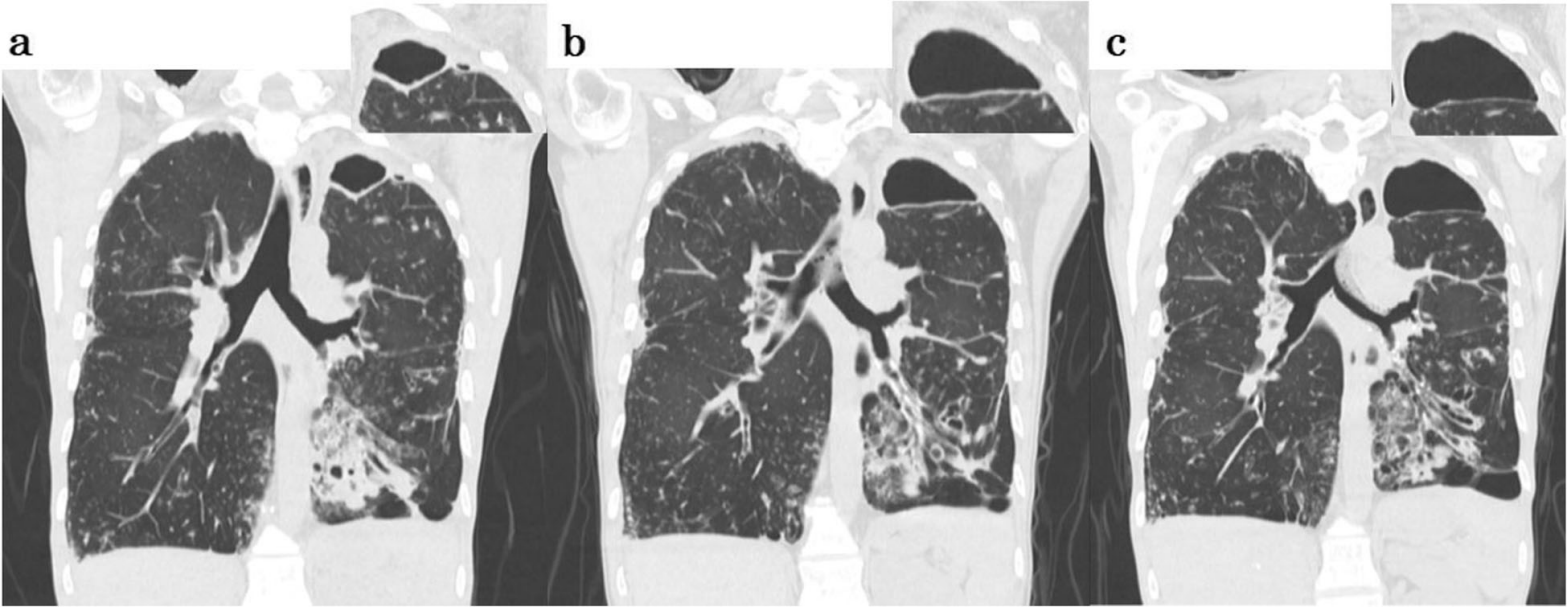
Comparison of the cavity lesion on coronal sections of high-resolution computed tomography. Images attached to the right shoulder are magnified views of cavity lesions. **a** A cavity lesion of the left lung apex was small at the first visit at our institution in 2014. **b** In 2017, the cavity lesion expanded and the wall thickened just before NTM treatment. **c** Repeat imaging in 2018, after NTM treatment, shows that the cavity wall is thinner

A 51-year-old woman had symptoms of cough and sputum for more than 10 years. Although the patient developed bronchiectasis at that time, it was not treated. Ten years later, in 2014, her symptoms of cough, sputum and breathlessness worsened, and she was referred to our hospital. As a medical history, she had more than 10 years of sinusitis. She did not have any relevant family history. At this initial visit, her height was 142.0 cm, body weight was 34.0 kg, and emaciation was remarkable. The patient did not have a fever, but a productive cough was heard during the examination. Coarse crackles were heard on auscultation. She had a part-time job in inventory despite a saturation of percutaneous oxygen (SpO_2_) rate of 91–92% at rest. Forced expiratory volume% in 1 s was dropped to 63.7%. A high-resolution computed tomography scan taken at the first consultation showed typical DPB findings, i.e., bilateral basal predominant bronchiectasis and centrilobular opacities with branching lines (Fig. 1). We diagnosed her with DPB, and she had HLA-B54 and HLA-A11. A cavity lesion in the apex of the left lung was also found (Fig. 2a). The sputum was acid-fast bacillus (AFB) smear-positive and culture-positive in liquid media after 6 days. Bronchoscopy was also performed, and bronchoalveolar lavage fluid was AFB smear-positive and culture-positive after 8 days. However, no NTM species corresponding to 17 types including *M.avium, M.intracellulare, M.kansasii, M.marinum, M.simiae, M.scrofulaceum, M.gordonae, M.szulgai, M.gastri, M.xenopi, M.nonchromogenicum, M.terrae, M.triviale, M.fortuitum, M.chelonae, M.abscessus, M.peregrinum* were identified with the DNA-DNA hybridization method. *P. aeruginosa* was also isolated from her sputum. She received treatment with long-term, low-dose erythromycin for DPB. Her condition had been stable, but the cavity enlarged and the cavity wall gradually thickened (Fig. 2a-b). Her sputum remained acid-fast bacillus smear-positive and culture-positive, which was considered to be a cause of the wall thickness. Respiratory samples were sent to the Research Institute of Tuberculosis, Japan Anti-tuberculosis Association to identify the mycobacterial species.

An isolate cultured on 2% Ogawa medium (Kyokuto Pharmaceuticals, Tokyo, Japan) was harvested and suspended in 500 μl of TE buffer. The suspension was boiled for 10 min for DNA extraction, centrifuged for 5 min at 12,000 xg, and 10 μl of the supernatant was used for PCR amplification of 16S rRNA (primer 285-rP2). The sequence was then analyzed by Applied Biosystems 3730xl DNA analyzer (Macrogen, Seoul, South Korea), and the result was subjected to BLAST homology search. The isolate showed 100% nucleotide homology to the 16S rRNA sequence of *M. talmoniae* type strain ATCC BAA-2683, while other species showed < 98% similarity. Thus, more than 2 years after her first visit, the patient was finally diagnosed with *M. talmoniae* infection at the end of 2016.

Susceptibility testing of the patient isolate was performed using BrothMIC NTM (Kyokuto Pharmaceuticals, Tokyo, Japan) which was the method based on the Clinical and Laboratory Standard Institute (CLSI) in 2003 and was commonly used in Japan. Table 1 showed some of the minimal inhibitory concentration (MIC) results for antimicrobials. It inferred to be susceptible to amikacin, clarithromycin, rifampicin and to be resistant to levofloxacin referring MAC in vitro MIC category decision by BrothMIC NTM. Levofloxacin was included in the BrothMIC NTM test instead of ciprofloxacin or moxifloxacin in Japan. Therefore, the patient was started on daily standard treatment for NTM (600 mg clarithromycin, 150 mg rifampicin, and 500 mg ethambutol). Approximately 5 months after the start of treatment, sputum was AFB smear-negative and culture-negative. She continued treatment for 2 years. The thickened wall of the giant cavity improved (Fig. 2c), and the SpO_2_ rate increased from 91 to 92% to 93–94%. Her productive cough and shortness of breath were improved, and she could take care of her parents. After 2 years of combination therapy, acid-fast bacillus smear and culture remained negative with every half year sputum inspection.

**Table 1:** Antimicrobial susceptibility of *Mycobacterium talmoniae* using broth microdilution MIC values of the patients respiratory sample

Antimicrobial	MIC(ug/ml)
Rifabutin	0.25
Rifampicin	1
Amikacin	2
Clarithromycin	0.25
Levofloxacin	16

## Discussion and conclusions

We encountered a case of *M. talmoniae* with DPB, in which standard combination therapy for NTM was effective. Clinical cases of *M. talmoniae* remain rare. Since *M. talmoniae* was first reported in 2017 [6], three previous isolations as clinical cases and only one clinical case requiring treatment of this NTM have been reported in the US to date [7]. In our case, NTM was found early in the clinical course, however, the species was not identified for 2.5 years.

NTM are environmental organisms that cause lung disease, especially in patients with pre-existing lung damage [17]. In our case, when the patient first visited another hospital in 2004, the cavity in the apex of the left lung was not detected. However, 10 years later, the cavity lesion appeared and gradually expanded 2 years later. In general, the local cavitary lesion in DPB is atypical. Kurashima et al. demonstrated that small nodules of MAC progress to cavities over a mean duration of 10 years in patients with middle lobe syndrome [18]. Therefore, in the present case, previous bronchiectatic lesions associated with DPB may have developed into the cavity lesion by NTM. Defects in the mucociliary transport system are assumed to be a predisposing factor to NTM infection, as observed in CF [19]. The first clinical case of *M. talmoniae* was detected in a CF patient [7]. Although DPB is more common than CF in East Asia, including Japan, reduced mucociliary clearance with bronchiectasis is an overlapping pathological condition in both diseases.

Previous papers on *M. talmoniae* was exceedingly few. The Davidson, et al. publication states that the *M. talmoniae* species shows highest similarity (only 97%) by 16S rRNA gene sequence to the type strain of *M. simie* and highest similarity (only 92%) to *M. avium* by the rpoB gene (less commonly used than 16S rRNA gene) [6]. According to their study, although antimicrobial susceptibility testing MIC values of *M. talmoniae* differed from the *M. avium* control strain, it showed similar values on clarithromycin, rifampin and ethambutol [6]. Another study reported three cases of *M. talmoniae* isolated from a human respiratory sample, and all three cases showed similar antimicrobial susceptibility which was susceptible to amikacin, clarithromycin, rifampin, and resistant to ciprofloxacin, linezolid, and minocycline [7]. Only one case with CF among them was treated by clarithromycin, rifampin, and ethambutol, and after 1 month of treatment her sputum became AFB smear and culture negative [7]. In our case, the *M. talmoniae* strain isolated from the patient’s respiratory samples was similarly susceptible to antimicrobials to clarithromycin, rifampicin and amikacin as seen in Table 1. Therefore, we treated the patient with standard combination therapy for NTM including clarithromycin, rifampicin, and ethambutol. Although symptoms of cough and sputum improved after low-dose erythromycin therapy, hypoxemia did not recover. However, after NTM treatment, both symptoms and clinical presentation, including the thickened giant cavity wall, improved. This result suggests that *M. talmoniae* was partially responsible for the patient’s clinical disease.

Although *M. talmoniae* infection remains uncommon, *M. talmoniae* should be considered as a potential pathogen of lung disease, especially in patients with bronchiectatic lesions.

## Data Availability

The classification reference databases were downloaded from National Center Biotechnology Information (https://www.ncbi.nlm.nih.gov/). The data used or analyzed in this case report and more detailed data are available from the corresponding author on reasonable request.

## Abbreviations

AFB: Acid-fast bacillus
CF: Cystic fibrosis
CLSI: Clinical and Laboratory Standards Institute
DPB: Diffuse panbronchiolitis
HLA: Human leukocyte antigen
*M*: *Mycobacterium*
MAC: *Mycobacterium avium* complex
MIC: Minimal inhibitory concentration
NTM: Non-tuberculous mycobacteria
SpO_2_: Saturation of percutaneous oxygen
US: The United States
